# Instagram, the new ally of Pro-Ana and Pro-Mia

**DOI:** 10.1192/j.eurpsy.2021.1859

**Published:** 2021-08-13

**Authors:** G. Lladó Jordan, M.D.C. Díaz García, P. Mediavilla Sánchez, B. Lozano Díez, J.A. Gómez Del Barrio, R. Ayesa-Arriola

**Affiliations:** Idival, Valdecilla Biomedical Research Institute, Santander, Spain

**Keywords:** Instagram, Pro-Ana, Pro-Mia, eating disorders

## Abstract

**Introduction:**

Nowadays Social Networks (SN) are used not only in a playful way but also as a ‘health’ means of communication. The Pro-Ana and Pro-Mia accounts or profiles -whereby Eating Disorders are advocated as a ‘lifestyle’- increased by 300% over the last decade.

**Objectives:**

To analyze Instagram Pro-Ana and Pro-Mia accounts and compare them with Pro-Ana and Pro-Mia Blogs.

**Methods:**

A non-computerized research of Pro-Ana and Pro-Mia Blogs and Instagram profiles was performed. Accepting a risk of Alpha=0.05 and Beta=0.15 in a two-tailed test, 29 subjects were required in each group to detect a difference equal to or greater than 0.2 units. The common standard deviation is assumed to be 0.25. Publication averages, photos, opening years, WhatsApp links and number of followers were analysed and compared. Transversal descriptive study.

**Results:**

Blogs: 100% had no groups in other SN, 33.33% had been opened for more than 3 years, 30% included personal pictures, 16.67% contained Ana in their title, 53.3% named other Blogs. Instagram: 56.67% included personal pictures, 13.33% mentioned WhatsApp groups, 73.33% had a public profile, 43.33% contained ‘Ana’ in their user name and 53.33% had more than one hundred followers.

**Conclusions:**

These tools are constantly adapting to the times in which they coexist. There has also been a current increase in Instagram profiles. This study shows a greater linkage to WhatsApp groups on Instagram than on Blogs, together with a higher number of followers, ease of ownership and difficulty of control.

**Disclosure:**

No significant relationships.

